# Prediction of intracranial findings on CT-scans by alternative modelling techniques

**DOI:** 10.1186/1471-2288-11-143

**Published:** 2011-10-25

**Authors:** Tjeerd van der Ploeg, Marion Smits, Diederik W Dippel, Myriam Hunink, Ewout W Steyerberg

**Affiliations:** 1 Medical Centre Alkmaar/Inholland University, Alkmaar, The Netherlands; 2 Erasmus Medical Centre, Rotterdam, The Netherlands

## Abstract

**Background:**

Prediction rules for intracranial traumatic findings in patients with minor head injury are designed to reduce the use of computed tomography (CT) without missing patients at risk for complications. This study investigates whether alternative modelling techniques might improve the applicability and simplicity of such prediction rules.

**Methods:**

We included 3181 patients with minor head injury who had received CT scans between February 2002 and August 2004. Of these patients 243 (7.6%) had intracranial traumatic findings and 17 (0.5%) underwent neurosurgical intervention. We analyzed sensitivity, specificity and area under the ROC curve (AUC-value) to compare the performance of various modelling techniques by 10 × 10 cross-validation. The techniques included logistic regression, Bayes network, Chi-squared Automatic Interaction Detection (CHAID), neural net, support vector machines, Classification And Regression Trees (CART) and "decision list" models.

**Results:**

The cross-validated performance was best for the logistic regression model (AUC 0.78), followed by the Bayes network model and the neural net model (both AUC 0.74). The other models performed poorly (AUC < 0.70). The advantage of the Bayes network model was that it provided a graphical representation of the relationships between the predictors and the outcome.

**Conclusions:**

No alternative modelling technique outperformed the logistic regression model. However, the Bayes network model had a presentation format which provided more detailed insights into the structure of the prediction problem. The search for methods with good predictive performance and an attractive presentation format should continue.

## Background

Minor head injury is one of the most common injuries seen in western emergency departments. Patients with minor head injury include those with blunt injury to the head who have a normal or minimally altered level of consciousness on presentation at the emergency department. Intracranial complications after minor head injury are infrequent, but they commonly require in-hospital observation and occasionally even neurosurgical intervention.

The imaging procedure of choice for reliable, rapid diagnostics of intracranial complications is computed tomography (CT). However, it is inefficient to scan all patients with minor head injury to exclude intracranial complications, as most patients with minor head injury do not show traumatic abnormalities on CT.

Several prediction rules have been developed to identify those at risk of abnormalities on CT. These include the CT in Head Injury Patients (CHIP) prediction rule [1], the Canadian CT Head Rule (CCHR) [2] and the New Orleans Criteria (NOC) [3]. While the NOC was developed by expert opinion and based on existing literature, the CCHR and CHIP rules were developed with recursive partitioning (Classification And Regression Trees, CART) and logistic regression techniques respectively (Table 1).

**Table 1:** Rules

Rule	Patient selection	N patients	N predictors considered	N predictors included	Modelling technique
NOC	Prospective cohort study	520	> 7	7	Expert opinion
CCHR	Prospective cohort study	3121	24	7	Logistic regression/CART
CHIP	Prospective cohort study	3181	14	14	Logistic regression
Lancet	Prospective cohort study	42411	10	3	CART

A recent study used CART modelling to develop a prediction rule for CT scanning in children [4]. CART modelling was argued to be a more appropriate method for the particular problem of selecting a very low risk group among patients with possible intracranial complications.

We hypothesized that alternative modelling techniques might deliver better results in terms of applicability and performance than modelling based on conventional modelling techniques such as logistic regression techniques. We compared logistic regression modelling to alternative modelling techniques [5,6], including CART and six other techniques, in the context of selective CT scanning for minor head injury. Data from the CHIP study, underlying the CHIP prediction rule, were used for this purpose.

## Methods

The CHIP database contains data on 3181 patients with minor head injury, defined as a presenting Glasgow Coma Scale (GCS) score of 13 to 15, and a maximum loss of consciousness of 15 minutes, posttraumatic amnesia for 60 minutes. Several risk factors were recorded to predict the presence of intracranial traumatic findings on CT (Table 2). Most of the risk factors were dichotomous variables (absent, present) and a few were continuous. The outcome of interest was intracranial traumatic findings on CT (absent, present). These intracranial traumatic findings included contusions, small hemorrhages indicating diffuse axonal injury, subarachnoid haemorrhage, and subdural and epidural hematoma, but excluded isolated linear skull fractures.

**Table 2:** Patient characteristics

		Intracranial lesions				
		***absent***		***present***		
		
		***n***	***(%)***	***n***	***(%)***	***p-value***

Fracture skull	Absent	2901	(98.7)	207	(85.2)	0.000
	Present	37	(1.3)	36	(14.8)	
EMV presentation (total) = 13	Absent	2818	(95.9)	212	(87.2)	0.000
	Present	120	(4.1)	31	(12.8)	
EMV presentation (total) = 14	Absent	2447	(83.3)	166	(68.3)	0.000
	Present	491	(16.7)	77	(31.7)	
Memory deficit	Absent	2535	(86.3)	171	(70.4)	0.000
	Present	403	(13.7)	72	(29.6)	
Contusion skull	Absent	1863	(63.4)	103	(42.4)	0.000
	Present	1075	(36.6)	140	(57.6)	
Loss of consciousness	Absent	1169	(39.8)	61	(25.1)	0.000
	Present	1769	(60.2)	182	(74.9)	
Seizure	Absent	2920	(99.4)	238	(97.9)	0.000
	Present	18	(0.6)	5	(2.1)	
Vomiting	Absent	2651	(90.2)	188	(77.4)	0.000
	Present	287	(9.8)	55	(22.6)	
Coumarins	Absent	2868	(97.6)	230	(94.7)	0.005
	Present	70	(2.4)	13	(5.3)	
Neurological deficit (all)	Absent	2676	(91.1)	201	(82.7)	0.000
	Present	262	(8.9)	42	(17.3)	
Cause	Reference	1882	(64.1)	102	(42)	0.000
	ped.or cyclist	297	(10.1)	51	(21)	
	Fall	702	(23.9)	82	(33.7)	
	Ejected	57	(1.9)	8	(3.3)	
PTA in 3 categories	< = 2 hrs.	2910	(99.0)	232	(95.5)	0.000
	> 2 hrs. and < = 4 hrs.	25	(0.9)	6	(2.5)	
	> 4 hrs.	3	(0.1)	5	(2.1)	
		***mean***	***(sd)***	***mean***	***(sd)***	***p-value***
EMV change		0.07	(0.50)	-0.04	(1.27)	0.186
Age - 16 per decade		2.48	(1.85)	3.22	(2.01)	0.000

Based on this set of predictors, the CHIP-prediction rule was previously developed for the identification of intracranial traumatic findings on CT, using logistic regression for the statistical modelling [1].

We compared the logistic regression model to alternative modelling techniques in developing prediction rules for intracranial findings on CT. We used the predictors listed in Table 2.

The following alternative modelling techniques were considered:

• Bayes network

• Neural net

• CHAID

• Support vector machine

• CART

• Decision list

### Description of the modelling techniques

The alternative modelling techniques compared in this study are briefly described below [7]

#### Bayes network

A Bayesian network is a graphical model that displays variables (often referred to as nodes) in a dataset and the probabilistic, or conditional, dependencies between them. Causal relationships between nodes may be represented by a Bayesian network; however, the links in the network (also known as arcs) do not necessarily represent direct cause and effect. For example, a Bayesian network can be used to calculate the probability of a patient having a specific disease, given the presence or absence of certain symptoms and other relevant data, if the probabilistic dependencies between symptoms and disease as displayed on the graph hold true. Networks are robust to missing information and aim to make the best possible prediction using whatever information is present.

There are several reasons to use a Bayesian network:

• It helps to learn about (potentially causal) relationships.

• The network provides an efficient approach to prediction by parsimonious modelling and aims to avoid overfitting of data.

• It offers a clear visualization of the relationships involved.

#### Neural net

A neural network, sometimes called a multilayer perceptron, is a simplified model of the way the human brain processes information. It works by simulating a large number of interconnected simple processing units that resemble abstract versions of neurons. The processing units are arranged in layers. There are typically three parts in a neural network: an input layer, with units representing the predictor variables, one or more hidden layers and an output layer, with a unit representing the outcome variable.

The units are connected with varying connection strengths or weights. Input data are presented to the first layer, and values are propagated from each neuron to every neuron in the next layer. Eventually, a prediction is delivered from the output layer. The network learns by examining individual records, generating a prediction for each record and making adjustments to the weights whenever it makes an incorrect prediction. This process is repeated many times, and the network continues to improve its predictions until one or more of the stopping criteria have been met.

With the default setting, the network will stop training when the network appears to have reached its optimally trained state (90% accuracy). The networks that fail to train well are discarded as training progresses.

Initially, all weights are random, and the predictions that come out of the net are nonsensical. The network learns through training. Records for which the output is known are repeatedly presented to the network, and the predictions it gives are compared to the known outcomes.

As training progresses, the network becomes increasingly accurate in replicating the known outcomes. Once trained, the network can be applied to future patients for whom the outcome is unknown.

#### CHAID

The Chi-squared Automatic Interaction Detection model is a classification method for building decision trees by using chi-square analysis to identify optimal splits. CHAID first examines the cross tables between each of the predictor variables and the outcome and tests for significance using a chi-square test. If more than one of these relations is statistically significant, CHAID will select the predictor that has the smallest p-value. If a predictor has more than two categories, these are compared, and categories that show a similar outcome are collapsed together. This is done by successively joining the pair of categories showing the least significant difference. This category-merging process stops when all remaining categories differ at the specified testing level. For set predictors, any categories can be merged. For an ordinal set, only contiguous categories can be merged. Exhaustive CHAID is a modification of CHAID that more thoroughly examines all possible splits for each predictor but takes longer to compute. CHAID can generate non-binary trees, meaning that some splits have more than two branches. It therefore tends to create a wider tree than the binary growing methods. CHAID works for all types of predictors.

#### Support vector machine

A Support Vector Machine (SVM) performs classification tasks by constructing hyperplanes in a multidimensional space that separates cases from different classes. It claims to be a robust classification and regression technique that maximizes the predictive accuracy of a model without overfitting the training data. A SVM may particularly be suited to analyze data with large numbers of predictor variables. SVM has applications in many disciplines, including customer relationship management (CRM), image recognition, bioinformatics, text mining concept extraction, intrusion detection, protein structure prediction, and voice and speech recognition.

#### CART

The Classification And Regression Tree model is a tree-based classification and prediction model. The model uses recursive partitioning to split the training records into segments with similar output variable values. The modelling starts by examining the input variables to find the best split, measured by the reduction in an impurity index that results from the split. The split defines two subgroups, each of which is subsequently split into two further subgroups and so on, until the stopping criterion is met.

#### Decision list

A Decision list model identifies subgroups or segments that show a higher or lower likelihood of a binary outcome relative to the overall sample. The model consists of a list of segments, each of which is defined by a rule that selects matching records. A given rule may have multiple conditions. Rules are applied in the order listed, with the first matching rule determining the outcome for a given record. Taken independently, rules or conditions may overlap, but the order of rules resolves ambiguity. If no rule matches, the record is assigned to the remainder rule.

### Cut-off values

For each model we determined cut-off values and classification rules to achieve a sensitivity > 0.95. To this end, we varied the cut-off values for each model from 0.015 to 0.05. Furthermore, the reduction in CT scans was calculated given a certain cut-off value. Reduction was defined as the percentage of subjects who would not undergo CT scanning since absence of intracranial findings on CT was predicted.

### Modelling

For the various modelling techniques we used Clementine Modeller version 12.0 in combination with SPSS 16.0. The comparison was made using performance characteristics including the area under the ROC curve, sensitivity and specificity. We used default modelling settings as far as possible (Additional file 1: Appendix 1). For the CART model, however, we used an extended setting besides the default setting. The stopping criteria for the default setting were: 100 records in the parent branch and 50 records in the child branch. The stopping criteria for the extended setting were: 11 records in the parent branch and 10 records in the child branch. In both variants we used pruning (Additional file 2: Appendix 2).

### Cross-validation

The models were validated using 10 × 10 cross-validation. The file was split into 10 random deciles. Each model was trained repeatedly on 9 deciles with predictions generated for the remaining decile. The AUC-values were calculated for the 10 training parts and the full set of 10 deciles which were left out of the training parts. The difference defined the optimism of each model, and this process was repeated 10 times. The optimism was subtracted from the apparent AUC-value for each model on the original sample to obtain optimism-corrected estimates of model performance [8].

## Results

### Comparison of the performance of the models

The logistic regression and CART models showed limited optimism in the AUC-values (< 0.040, Table 3). The support vector machine model had a remarkably high optimism (0.171). The logistic regression model had the best performance (optimism-corrected AUC 0.787), followed by the Bayes network model (AUC 0.744) and the neural net model (AUC 0.726). The CHAID model and the decision list model had AUC-values of 0.699 and 0.634 respectively. The support vector machine model and the default CART model performed poorly with AUC-values 0.581 and 0.560 respectively. Although the CHAID model was more overfitted, the optimism-corrected AUC-value was much better than the CART analyses (Table 3).

**Table 3:** AUC-values

Model	AUC	95% CI for AUC	Mean AUC training	Mean AUC test	Optimism	Optimism-Corrected AUC
Logistic regression	0.800	0.769 - 0.830	0.789	0.772	0.017	0.783
Neural net	0.782	0.751 - 0.814	0.785	0.746	0.038	0.744
Bayes network	0.806	0.777 - 0.836	0.808	0.743	0.065	0.741
CHAID	0.759	0.724 - 0.794	0.761	0.686	0.075	0.684
Decision list	0.674	0.633 - 0.715	0.673	0.626	0.048	0.627
CART extended	0.657	0.616 - 0.699	0.599	0.559	0.040	0.617
Support vector machine	0.754	0.714 - 0.794	0.740	0.578	0.162	0.592
CART default	0.568	0.527 - 0.609	0.556	0.537	0.019	0.549

The default CART model showed less statistical optimism than the extended CART model (0.008 versus 0.039 respectively). However, the optimism-corrected AUC-value was worse for the default CART model (AUC 0.560 versus 0.618 respectively, Table 3).

The logistic regression model had a sensitivity of 0.98 and a reduction of 20% at a cut off value of 0.02. The Bayes network model had a sensitivity of 0.97 and a reduction of 23% at a cut off value of 0.015. For the neural net model, it was not possible to achieve a sensitivity > 0.95.

### Graphical representations

**Figure 1:**
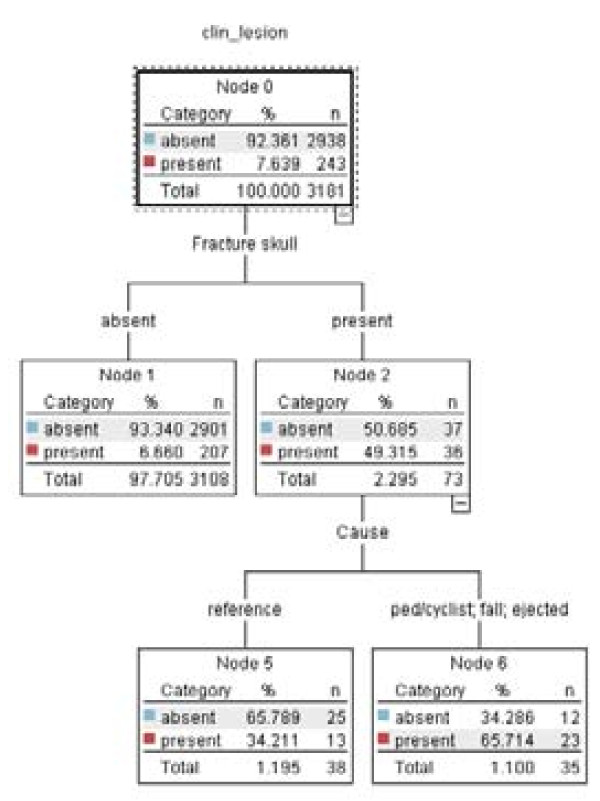
**CART model default**.

**Figure 2:**
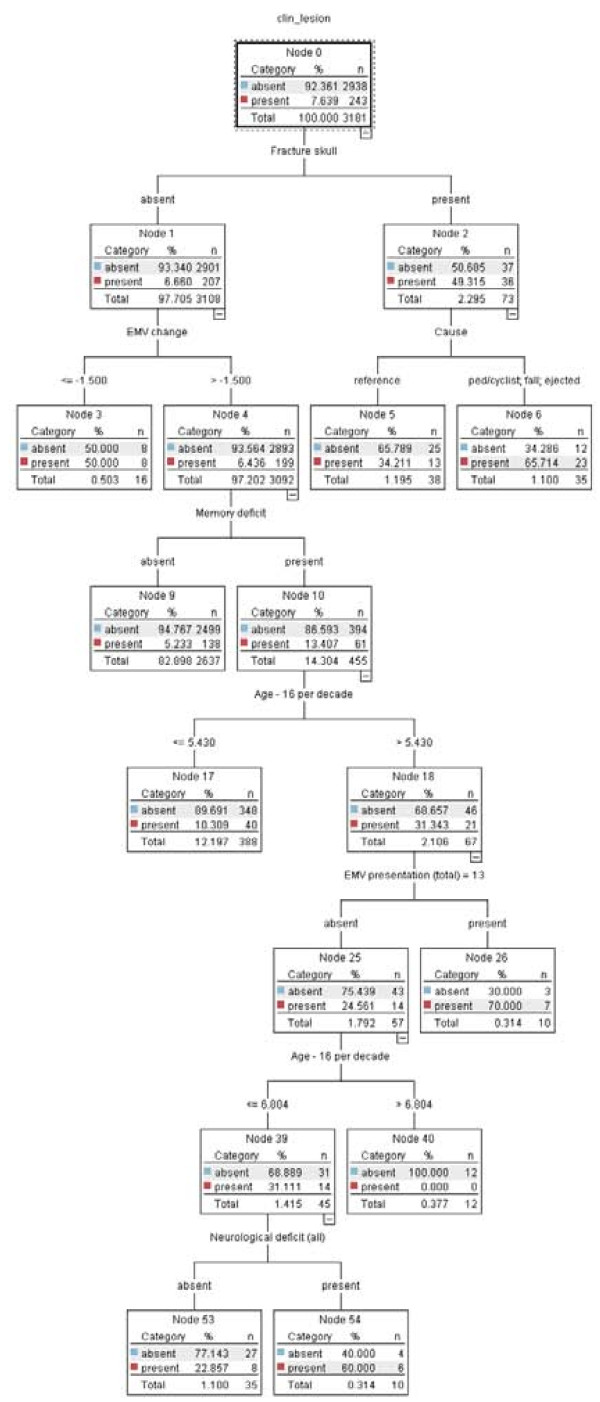
**CART model extended**.

The CART model is presented as a tree. The default CART model consisted of two predictor variables (Fracture skull and Cause), which were presented with three end nodes (Figure 1). The extended CART model consisted of six predictor variables (Fracture skull, EMV change, Cause, Memory deficit and Age per decade) presented in a tree with nine end nodes (Figure 2).

**Figure 3:**
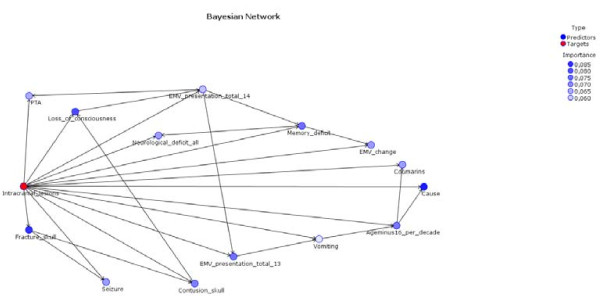
**Bayesian network model**.

The Bayes network model is presented an interaction graph. It shows the relative importance of the predictors (Figure 3). The variable 'intracranial lesions' had a direct relation with the variable 'fracture skull' and the variable 'seizure'. It also showed a relation between the variable 'fracture skull' and the variable 'seizure'.

**Figure 4:**
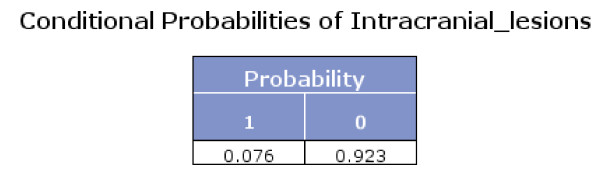
**Conditional probabilities of Intracranial lesions**.

**Figure 5:**
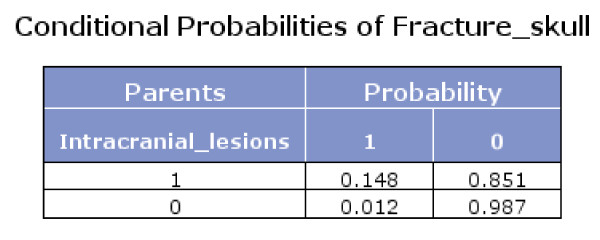
**Conditional probabilities of Fracture skull**.

**Figure 6:**
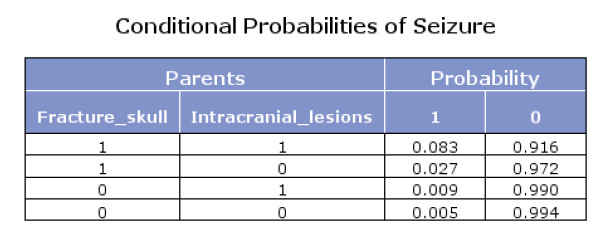
**Conditional probabilities of Seizure**.

The Bayes network model also presented the conditional probabilities (Figures 4, 5 and 6). Figure 6 shows that if fracture skull is absent and intracranial lesions are absent, the probability that seizure is absent equals 0.994.

**Figure 7:**
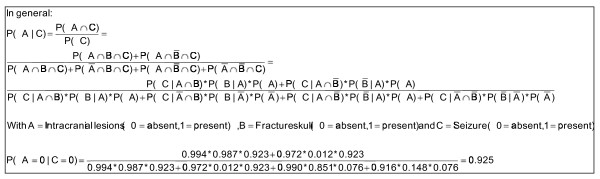
**Calculation example**.

Using Bayes theorem and the conditional probabilities in the figures 4, 5 and 6, we calculated that if seizure is absent, the predicted probability that intracranial traumatic findings are absent equals 92.5% (Figure 7).

**Figure 8:**
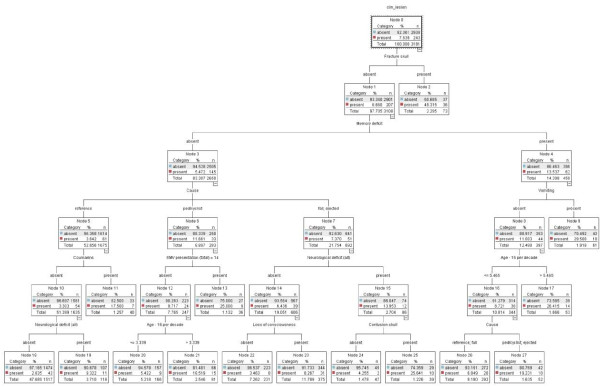
**CHAID model**.

The CHAID model presented a tree graph. The tree consisted of fifteen end nodes and therefore of fifteen decision rules (Figure 8). Hence the tree size was much larger than that of the CART analyses (Figure 1 and Figure 2).

### Presentation of the logistic regression model

The coefficients of the logistic regression model are presented in Table 4. The probabilities were calculated using Formula 1.

**Table 4:** Regression coefficients logistic model

Variables	X	b
Fracture skull	Present	2.34
	Absent	0.00
EMV presentation (total) = 13	Present	1.37
	Absent	0.00
EMV presentation (total) = 14	Present	0.72
	Absent	0.00
Memory deficit	Present	0.41
	Absent	0.00
Contusion skull	Present	0.59
	Absent	0.00
Loss of consciousness	Present	0.60
	Absent	0.00
Seizure	Present	0.84
	Absent	0.00
Vomiting	Present	0.88
	Absent	0.00
Coumarins	Present	0.87
	Absent	0.00
Neurological deficit (all)	Present	0.40
	Absent	0.00
EMV change	EMV change	-0.32
Cause	Reference	0.00
	pedastrian or cyclist	1.27
	Fall	0.55
	Ejected	1.13
Age - 16 per decade	Age - 16 per decade	0.17
PTA	< = 2 hrs	0.00
	> 2 hrs and < = 4 hrs	0.48
	> 4 hrs	2.01
Constant	Constant	-4.77

Formula 1 Calculation probabilities logistic regression model (π)

$$\text{π}=\frac{1}{1+{\text{e}}^{-\sum {\text{X}}_{\text{i}}*{\text{b}}_{\text{i}}}}$$

## Discussion

We found that alternative modelling techniques did not deliver better results in terms of applicability and performance in developing prediction rules for intracranial findings in patients with minor head injury than modelling based on conventional modelling techniques such as logistic regression. The performance of logistic regression was compared with six alternative modelling techniques using standard measures, specifically the receiver operating characteristic (ROC) curve. In a ROC curve, the trade-off between sensitivity and specificity is shown based on consecutive cut-off values. The key characteristic for model comparisons is the area under the ROC curve, which is equivalent to the concordance (or 'c') statistic.

The apparent AUC-values of each model were corrected for optimism using 10 × 10 cross-validation. Only the logistic regression model, the Bayes network model and the neural net model had satisfactory AUC-values (> 0.7), although it was impossible to achieve a sensitivity > 0.95 for the neural net model. The CHAID model and the decision list model had AUC-values of 0.699 and 0.634 respectively, and the support vector machine model and the default CART model performed poorly (AUC-values < 0.6).

At a cut-off value of 0.015, the logistic regression model would miss only 1% of the patients with intracranial traumatic findings (sensitivity 99%), whereas the Bayes network model would miss 3% (sensitivity 97%) at this cut-off. On the other hand, at this cut-off value the specificity of the Bayes model would be better (25%), and could potentially reduce the number of CT scans ordered by 23%. In contrast, the logistic regression model would only have 8% specificity and would reduce the number of CT scans ordered by 8% at a cut-off of 0.015. This illustrates the difficult trade-off between missing patients with intracranial traumatic findings versus the wish to reduce unnecessary CT scans in those without intracranial traumatic findings.

No modelling technique outperformed the relatively simple logistic regression model in terms of the optimism-corrected AUC-value. These findings may be seen as confirming the validity of the previously developed CHIP prediction rule [1]. However, it should be noted that these results are an internal validation of the developed CHIP-rule and that external validation is still required.

Our findings are in contrast to a recent study that advocated CART modelling to develop a prediction rule for CT scanning in children [4]. This can potentially be explained by the fact that modelling techniques such as CART are 'data hungry'. Therefore CART modelling may have been suitable for the Kuppermann study, which included 42,411 patients (376 with abnormal CT scans). However, it was not suitable for the CHIP database, which included only 3,181 patients (243 with abnormal CT scans). Also, the specific algorithm used in the Kuppermann study may have been different from the algorithm used in our study.

The superior performance of the logistic regression modelling might be explained by the high number of categorical variables (10 out of 14), which might favour logistic regression modelling. The somewhat disappointing performance of tree models like CHAID and CART may be more realistic, because these models are well suited for dealing with categorical and continuous variables, although the latter are categorized by these models.

Although the examined modelling techniques did not outperform logistic regression analysis, we can see a role for these techniques in providing a deeper insight into the interrelationships between predictors and outcome. For example, the Bayes network offered the advantage of showing a graphical representation of the direct relationships between the predictor variables and the outcome variable, as well as the first-order interactions. The CHAID model offered a tree graph which might give researchers insight into relevant risk groups. The neural net model, on the other hand, did have a satisfactory optimism-corrected AUC-value, but did not provide further insight into the medical problem. This alternative modelling technique has a black box character, which is a serious drawback for application in medical practice.

The outcomes of this study suggest that the use of alternative modelling techniques may also have practical value in ascertaining variables of critical import and in streamlining current existing guidelines. Smits et al. used 14 variables for their modelling based on expert opinion and previous studies. We started out with these same 14 variables to be able to compare the model of Smits et al. with modelling based on alternative modelling techniques. However, the CHAID model only used 10 out of these 14 variables. The variables PTA, Change, EMV-13 and Seizure were not used, which suggests that these variables may be of lower importance for the outcome. However, the CHAID model performed poorly in comparison with logistic regression modelling. For most of the evaluated models, the variables of critical import were: Fracture skull (v69), Cause (cause3) and Age - 16 per decade (age10). Based on our study, the guidelines should certainly contain these variables.

A priori, it is not fully predictable whether an alternative modelling technique will perform better than conventional modelling techniques. This depends on the internal structure of the prediction problem and on the characteristics of the modelling techniques. For example, tree modelling is well suited for a situation with many interactions between predictors, which might be missed with a default main effects logistic model. Neural nets are even more flexible in capturing interactions and non-linearities, which might be missed by other modelling techniques. It has been suggested that the balance between signal and noise is relatively unfavourable in many medical applications, making relatively simple regression models perform quite reasonably [9].

All these models can easily be evaluated, because capacity limitations for computer calculations no longer exist nowadays. The required software for evaluating the performance of alternative modelling techniques is readily available (e.g. Clementine, R software, etc). The methods we used in this study may be applied to other studies using characteristics such as AUC-values, sensitivity and specificity. Internal validation can be performed using 10 × 10 cross-validation. From there, optimism-corrected AUC-values can readily be calculated.

Depending on the software used, it is possible to use the default setting or to choose an expert setting for the CART modelling. A researcher may use an expert setting for the number of levels below the root of a tree, for the number of records in the parent node and the child node, for applying or not applying pruning, for using weights for the categories of the outcome variable (costs) and so on. In our study, we used the default settings for the modelling as far as possible. Only in the evaluation of the CART model did we use an extended setting besides the default setting in order to achieve a higher AUC-value, but even then the performance of this model was poor.

In view of the applicability and simplicity of a prediction model, medical experts and researchers usually prefer a small number of predictors. However, this study shows that a considerable number of variables may be necessary to make an informed decision or a prediction with a high level of accuracy. The CHIP rule included 14 variables as major and minor risk factors, which all turned out to be indispensible.

By comparison, the default CART model appeared attractive, as it consisted of only 3 end nodes and therefore of 3 decision rules. Unfortunately, this model showed a poor performance.

Larger models may lead to better performance when all predictors are in fact predictive of the outcome [10]. While the number of predictors should therefore not be unduly limited, the applicability and simplicity of a decision rule might still be improved by using a model that provides a clarifying presentation of all the relevant variables and their mutual dependencies. Therefore the search for superior models with attractive presentation formats should continue.

## Conclusions

No alternative modelling technique outperformed the logistic regression model. However, the Bayes network model had a presentation format which provided more detailed insights into the structure of the prediction problem. The search for methods with good predictive performance and an attractive presentation format should continue.

## Competing interests

The authors declare that they have no competing interests.

## Authors' contributions

TVDP conceived of the study, carried out the analyses and comparison of the modelling techniques and drafted the manuscript. MS, DWD and MH contributed the data and background information for the CHIP prediction rule. EWS participated in the design of the study and helped to draft the manuscript. All authors read and approved the final manuscript.

## Pre-publication history

The pre-publication history for this paper can be accessed here:

http://www.biomedcentral.com/1471-2288/11/143/prepub

## Supplementary Material

Additional file 1 **Appendix 1**. Modelling settings Click here for file

Additional file 2 **Appendix 2**. Characteristics of the models Click here for file

## References

[B1] Smits M Dippel DWJ Steyerberg EW De Haan GG Dekker HM Vos PE Kool DR Nederkoorn PJ Hofman PAM Twijnstra A Tanghe HLJ Hunink MGM Predicting Intracranial Traumatic Findings on Computed Tomography in Patients with Minor Head Injury: The CHIP Prediction Rule Annals of Internal Medicine 2007 146 6 397 405 17371884 10.7326/0003-4819-146-6-200703200-00004

[B2] Stiell IG Wells GA Vandemheen K Clement C Lesiuk H Laupacis A McKnight RD Verbeek R Brison R Cass D Eisenhauer ME Greenberg G Worthington J The Canadian CT Head Rule for Patients with Minor Head Injury The Lancet 2001 357 9266 1391 1396 10.1016/S0140-6736(00)04561-X 11356436

[B3] Stiell IG Clement CM Rowe BH Schull MJ Brison R Cass D Eisenhauer MA McKnight RD Bandiera G Holroyd B Lee JS Dreyer J Worthington JR Reardon M Greenberg G Lesiuk H MacPhail I Wells GA Comparison of the Canadian CT Head Rule and the New Orleans Criteria in Patients with Minor Head Injury JAMA 2005 28;294 12 1511 8 16189364 10.1001/jama.294.12.1511

[B4] Kuppermann N Holmes JF Dayan PS Hoyle JD Atabaki SM Holubkov R Nadel FM Monroe D Stanley RM Borgialli DA Badawy MK Schunk JE Quayle KS Mahajan P Lichenstein R Lillis KA Tunik MG Jacobs ES Callahan JM Gorelick H Glass TF Lee LK Bachman MC Cooper A Powell EC Gerardi MJ Melville KA Muizelaar JP Wisner DH Zuspan SJ Dean JM Wootton-Gorges SL Identification of children at very low risk of clinically-important brain injuries after head trauma: a prospective cohort study The Lancet 2009 374 9696 1160 1170 10.1016/S0140-6736(09)61558-0 19758692

[B5] Breiman L Friedman JH Olshen RA Stone CJ Classification and regression trees 1984 Boca Raton, Chapman & Hall/CRC

[B6] Breiman L Stone CJ Parsimonious binary classification trees 1978 Santa Monica, Technology Service Corporation

[B7] Clementine® 7.0 User's Guide 2002 Integral Solutions Limited

[B8] Steyerberg EW Clinical Prediction Models: A Practical Approach to Development, Validation, and Updating 2008 New York, Springer

[B9] Zani S Cerioli A Riani M Vichi M Data analysis, classification and the forward search: proceedings of the Meeting of the Classification and Data Analysis Group (CLADAG) of the Italian Statistical Society, University of Parma, June 6-8, 2005 2006 New York, Springer

[B10] Harrel FE Regression Modeling Strategies 2001 New York, Springer

