# Supplementary material for: Prediction of intracranial findings on CT-scans by alternative modelling techniques
Source: BMC Med Res Methodol. 2011 Oct 25;11:143. doi: 10.1186/1471-2288-11-143 (PMC3212831; doi:10.1186/1471-2288-11-143)
Supplement: Additional file 1 — Appendix 1. Modelling settings [file 1471-2288-11-143-S1.DOC]

**ADDITIONAL FILE 1**

**Appendix 1**

**Modelling settings**

| **Bayes network** |  |
| --- | --- |
| Build Settings |  |
|  | Use partitioned data: false |
|  | Variable importance.LABEL: true |
|  | Calculate raw propensity scores: true |
|  | Calculate adjusted propensity scores: false |
|  | Use frequency field: false |
|  | Continue training existing model: false |
|  | Structure type: TAN |
|  | Include feature selection preprocessing step: false |
|  | Parameter learning method: Maximum likelihood |
|  | Mode: Simple |
|  | Use only complete records: true |
|  | Append all probabilities: false |
|  | Independence test: Likelihood ratio |
|  | Significance level: 0,01 |
|  | Maximal conditioning set size: 5 |
|  | Inputs always selected: [] |
|  | Maximum number of inputs: 10 |
|  |  |
|  |  |
| **Neural net** |  |
| Build Settings |  |
|  | Use partitioned data: false |
|  | Calculate variable importance: true |
|  | Calculate raw propensity scores: true |
|  | Calculate adjusted propensity scores: false |
|  | Method: Quick |
|  | Stop on: Default |
|  | Set random seed: true |
|  | Set random seed: true |
|  | Prevent overtraining: false |
|  | Sample %: 50,0 |
|  | Optimize: Memory |
|  | Mode: Simple |
| Analysis |  |
|  | Estimated accuracy: 93,587 |
|  | Input Layer: 29 neurons |
|  | Hidden Layer 1: 3 neurons |
|  | Output Layer: 1 neurons |
|  |  |
|  |  |
| **CHAID** |  |
| Analysis |  |
|  | Tree depth: 5 |
| Build Settings |  |
|  | Use partitioned data: false |
|  | Calculate variable importance: true |
|  | Calculate raw propensity scores: true |
|  | Calculate adjusted propensity scores: false |
|  | Use frequency: false |
|  | Use weight: false |
|  | Levels below root: 5 |
|  | Mode: Simple |
|  | Use misclassification costs: false |
|  |  |
|  |  |
| **Support vector machine** |  |
| Build Settings |  |
|  | Use partitioned data: false |
|  | Variable importance.LABEL: true |
|  | Calculate raw propensity scores: true |
|  | Calculate adjusted propensity scores: false |
|  | Mode: Simple |
|  | Append all probabilities (valid only for categorical targets): false |
|  | Stopping criteria: 1.0E-3 |
|  | Kernel type: RBF |
|  | Regularization parameter (C): 10 |
|  | Regression precision (epsilon): 0,1 |
|  | RBF gamma: 0,1 |
|  | Gamma: 1,0 |
|  | Bias: 0,0 |
|  | Degree: 3 |
|  |  |
|  |  |
| **CART default** |  |
| Analysis |  |
|  | Tree depth: 2 |
| Build Settings |  |
|  | Use partitioned data: false |
|  | Calculate variable importance: true |
|  | Calculate raw propensity scores: true |
|  | Calculate adjusted propensity scores: false |
|  | Use frequency: false |
|  | Use weight: false |
|  | Levels below root: 10 |
|  | Mode: Simple |
|  | Use misclassification costs: false |
|  |  |
|  |  |
| **CART extended** |  |
| Build Settings |  |
|  | Use partitioned data: false |
|  | Calculate variable importance: true |
|  | Calculate raw propensity scores: true |
|  | Calculate adjusted propensity scores: false |
|  | Use frequency: false |
|  | Use weight: false |
|  | Levels below root: 5 |
|  | Mode: Expert |
|  | Maximum surrogates: 5 |
|  | Minimum change in impurity: 0,0 |
|  | Impurity measure for categorical targets: Gini |
|  | Stopping criteria: Use absolute value |
|  | Minimum records in parent branch: 11 |
|  | Minimum records in child branch: 10 |
|  | Prune tree: true |
|  | Use standard error rule: false |
|  | Prior probabilities: Based on training data |
|  | Adjust priors using misclassification costs: false |
|  | Use misclassification costs: false |
|  |  |
|  |  |
| **Decision list** |  |
| Build Settings |  |
|  | Use partitioned data: false |
|  | Calculate raw propensity scores: true |
|  | Calculate adjusted propensity scores: false |
|  | Use frequency: false |
|  | Target value: 1,0 |
|  | Search direction: Up |
|  | Maximum number of segments: 5 |
|  | Minimum segment size (as percentage): 5,0 |
|  | Minimum segment size (as absolute value): 50 |
|  | Maximum number of attributes: 5 |
|  | Allow attribute re-use: true |
|  | Confidence interval for new conditions (%): 95,0 |
|  | Mode: Simple |
|  |  |
|  |  |
| **Logistic regression** |  |
| Build Settings |  |
|  | Use partitioned data: false |
|  | Calculate variable importance: true |
|  | Calculate raw propensity scores: true |
|  | Procedure: Multinomial |
|  | Base category: 0 |
|  | Model type: Main Effects |
|  | Include constant in equation: true |
|  | Mode: Simple |
|  | Multinomial Method: Enter |
