# Supplementary material for: Prediction of intracranial findings on CT-scans by alternative modelling techniques
Source: BMC Med Res Methodol. 2011 Oct 25;11:143. doi: 10.1186/1471-2288-11-143 (PMC3212831; doi:10.1186/1471-2288-11-143)
Supplement: Additional file 2 — Appendix 2. Characteristics of the models [file 1471-2288-11-143-S2.DOC]

**ADDITIONAL FILE 2**

**Appendix 2**

**Characteristics of the models**

|  |  | **Model** | | | | | |
| --- | --- | --- | --- | --- | --- | --- | --- |
|  |  | Bayes network | CHAID and CART | Decision list | Support vector machine | Neural net | Logistic regression |
| Categorizing of continuous predictor variables | Yes | x | x | x |  | x |  |
|  | No |  |  |  | x |  | x |
| Outcome | Continuous |  | x |  | x | x |  |
|  | Categorial | x | x |  | x | x |  |
|  | Dichotomous | x | x | x | x | x | x |
| Iteractions | Assumed |  | x | x |  | x |  |
|  | Flexible | x |  |  | x |  |  |
|  | Possible |  |  |  |  |  | x |
| Selection of predictor variables | Assumed | x | x | x |  |  |  |
|  | Flexible |  |  |  | x | x | x |
| Graphical output | Tree graph |  | x |  |  |  |  |
|  | Interaction graph | x |  |  |  |  |  |
|  | Variable importance | x | x |  | x | x |  |
| Formula | Yes |  |  |  |  |  | x |
|  | No | x | x | x | x | x |  |
